# Inhibition of endothelial Cdk5 reduces tumor growth by promoting non-productive angiogenesis

**DOI:** 10.18632/oncotarget.6842

**Published:** 2016-01-08

**Authors:** Henriette Merk, Siwei Zhang, Thorsten Lehr, Christoph Müller, Melanie Ulrich, James A. Bibb, Ralf H. Adams, Franz Bracher, Stefan Zahler, Angelika M. Vollmar, Johanna Liebl

**Affiliations:** ^1^ Department of Pharmacy, Pharmaceutical Biology, Ludwig-Maximilians-University, 81377 Munich, Germany; ^2^ Clinical Pharmacy, Saarland University, 66123 Saarbrücken, Germany; ^3^ Department of Pharmacy, Pharmaceutical Chemistry, Ludwig-Maximilians-University, 81377 Munich, Germany; ^4^ Department of Psychiatry and Neurology and Neurotherapeutics, The University of Texas Southwestern Medical Center, Dallas, Texas 75390–9070, USA; ^5^ Department of Tissue Morphogenesis, Max Planck Institute for Molecular Biomedicine, 48149 Münster, Germany; ^6^ University of Münster, Faculty of Medicine, 48149 Münster, Germany

**Keywords:** Cdk5, angiogenesis, cancer, Notch

## Abstract

Therapeutic success of VEGF-based anti-angiogenic tumor therapy is limited due to resistance. Thus, new strategies for anti-angiogenic cancer therapy based on novel targets are urgently required. Our previous *in vitro* work suggested that small molecule Cdk5 inhibitors affect angiogenic processes such as endothelial migration and proliferation. Moreover, we recently uncovered a substantial role of Cdk5 in the development of lymphatic vessels. Here we pin down the *in vivo* impact of endothelial Cdk5 inhibition in angiogenesis and elucidate the underlying mechanism in order to judge the potential of Cdk5 as a novel anti-angiogenic and anti-cancer target. By the use of endothelial-specific Cdk5 knockout mouse models and various endothelial and tumor cell based assays including human tumor xenograft models, we show that endothelial-specific knockdown of Cdk5 results in excessive but non-productive angiogenesis during development but also in tumors, which subsequently leads to inhibition of tumor growth. As Cdk5 inhibition disrupted Notch function by reducing the generation of the active Notch intracellular domain (NICD) and Cdk5 modulates Notch-dependent endothelial cell proliferation and sprouting, we propose that the Dll4/Notch driven angiogenic signaling hub is an important and promising mechanistic target of Cdk5. In fact, Cdk5 inhibition can sensitize tumors to conventional anti-angiogenic treatment as shown in tumor xenograft models. In summary our data set the stage for Cdk5 as a drugable target to inhibit Notch-driven angiogenesis condensing the view that Cdk5 is a promising target for cancer therapy.

## INTRODUCTION

Inhibition of angiogenesis has shown clinical efficacy and represents a valid approach in cancer therapy. Patients benefit from the combination of chemotherapeutics or radiation with angiogenesis inhibitors. The VEGF pathway is currently the predominant target for anti-angiogenic therapy and anti-VEGF biologics (Bevacizumab) or small molecule inhibitors (Sorafenib, Sunitinib) are given clinically [1]. Unfortunately, non-responsiveness or resistance to anti-angiogenic treatment and subsequent tumor recurrence and metastasis limit therapeutic success [2]. Thus, finding new strategies for anti-angiogenic therapy represents an important and challenging objective in cancer research.

In this context, the Dll4/Notch pathway has emerged as an interesting target. In physiological angiogenesis, Dll4/Notch signaling regulates VEGF-induced vessel sprouting and branching and defines tip and stalk cell specification [3]. In tumors, activation of the Notch pathway promotes tumor growth [4] and mediates resistance to chemotherapy [5]. Of note, the disruption of Dll4/Notch signaling results in inhibition of tumor growth [4–8] and was associated with excessive but non-productive angiogenesis and impaired tumor vessel perfusion [6, 7]. Consequently, the blockade of the Dll4/Notch pathway is considered as a promising option for anti-angiogenic treatment. In fact, γ-secretase inhibitors or anti-Dll4/anti-Notch biologics are currently being evaluated in open clinical trials for cancer therapy [9, 10]. Furthermore, preclinical models indicated that the combination of targeting the Dll4/Notch pathway and anti-VEGF treatment leads to synergistic tumor growth inhibitory effects [5, 8]. However, little is known about the regulation of Dll4/Notch in tumor angiogenesis [9]. Moreover, the chemosensitization by combination therapies to overcome resistance to anti-angiogenic treatment demands more attention in order to develop new treatment strategies.

In this respect the protein kinase cyclin dependent kinase 5 (Cdk5) represents a particular interesting potential target to explore. Cdk5 is a serine/threonine kinase that is highly expressed in the central nervous system (CNS) and is essential for neuronal development and function [11–13], but its role in the periphery and in cancer is not well explored. During the recent years, the awareness about roles of Cdk5 besides the CNS has grown. Cdk5 is expressed in various non-neuronal tissues [14–16] and has been implicated in various types of cancer including pancreatic [17–19], prostate [20, 21], thyroid [22, 23], glioma [24], pituitary [25], breast [26], lung [27], ovarian [28], and hepatocellular [29] cancers affecting various targets such as retinoblastoma protein and downstream cell cycle regulators [22, 23], the PIKE-A-Akt pathway [24], Ras-Ral signaling [17], or DNA damage response [29]. Further, by applying cell-based assays, our former studies demonstrated that small molecule Cdk5 inhibitors exert anti-angiogenic properties [30, 31] and that Cdk5 regulates endothelial cell migration [32] which was restricted to *in vitro* assays. However, to nail down the *in vivo* significance of Cdk5 in the endothelium, we have recently generated constitutive and inducible endothelial-specific Cdk5 knockout mouse models, elucidating an indispensable requirement of Cdk5 for lymphatic vessel development and function [33].

Here, by using the endothelial-specific Cdk5 knockout mouse models, endothelial and tumor cells, and human tumor xenografts, we investigate the heretofore unknown *in vivo* function of Cdk5 in the blood vessel endothelium. Moreover, the contribution of endothelial Cdk5 to tumor angiogenesis and the underlying mechanism such as the Dll4/Notch driven angiogenic signaling are important subjects of this work.

## RESULTS

### Inhibition of Cdk5 in the endothelium induces hypervascularization

**Figure 1:**
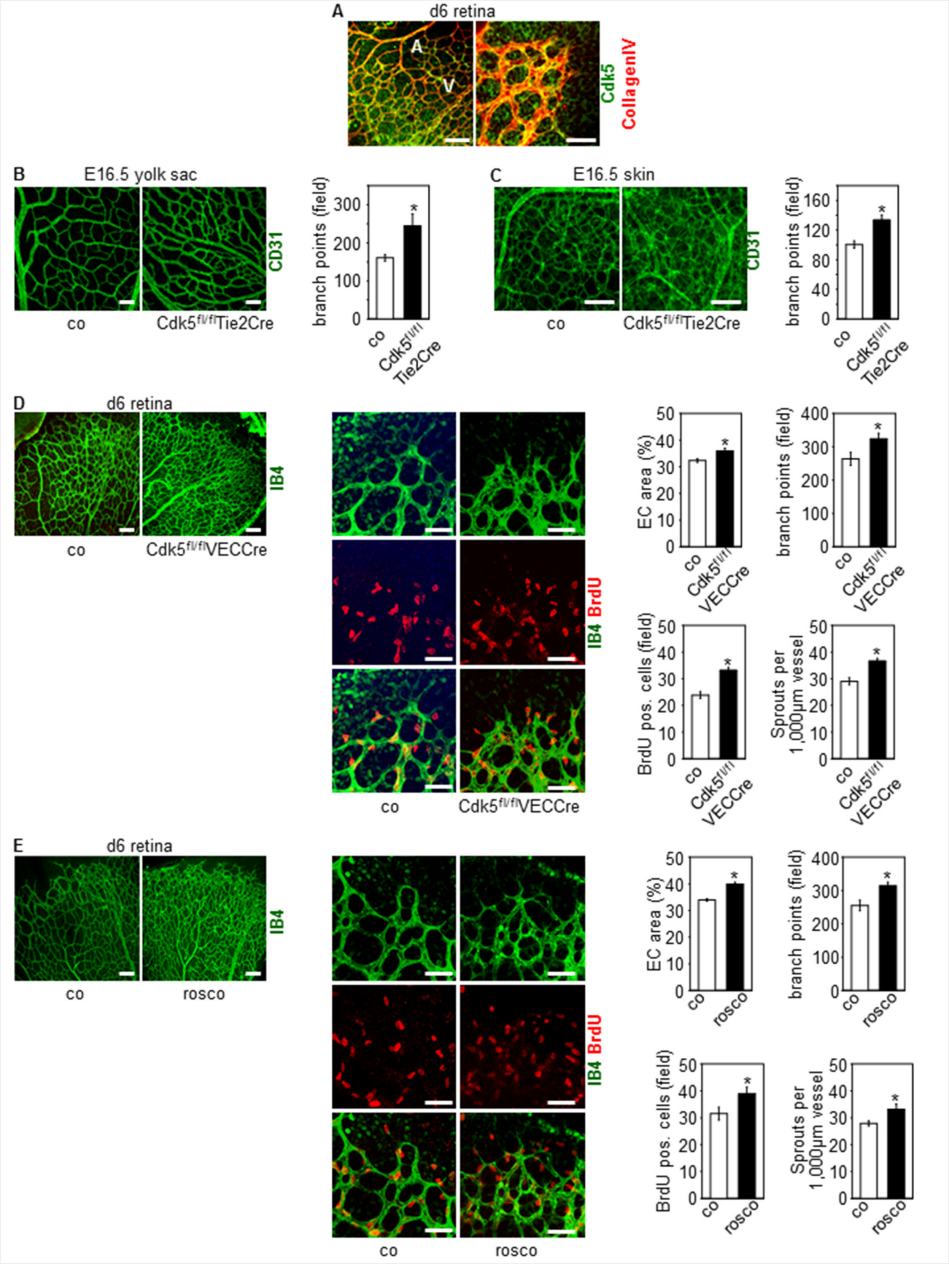
Knockdown and pharmacological inhibition of Cdk5 in the endothelium induces hypervascularization (**A**) Expression of Cdk5 in the mouse endothelium is shown by immunostainings of the developing retina (d6) for Cdk5 (green) and collagen IV (red). Arteries (A) and veins (V) (left panel) are indicated. *n* = 3. Scale bar (left panel) 100 μm. Scale bar (right panel) 50 μm. (**B**) CD31 stainings (green) of yolk sacs of E16.5 embryos with control and Cdk5^fl/fl^Tie2Cre genotype are shown. Scale bar 100 μm. Quantification of branching points is displayed. *t*-test, SEM, *p = 0.023, control: *n* = 13; Cdk5^fl/fl^Tie2Cre: *n* = 5. (**C**) CD31 stainings (green) of skin of E16.5 embryos with control and Cdk5^fl/fl^Tie2Cre genotype are shown. Scale bar 100 μm. Quantification of branching points is displayed. *t*-test, SEM, *p = 0.004, control: *n* = 9; Cdk5^fl/fl^Tie2Cre: *n* = 5. (**D**) Isolectin B4 staining (IB4, green) and BrdU labeling (red) of retinae from control (*n* = 8) and Cdk5^fl/fl^VECCre (*n* = 10) pups (d6) is shown. Scale bars (upper panels) 100 μm. Scale bars (lower panels) 50 μm. Quantifications of the area covered by ECs (*t*-test, SEM, *p = 0.015), the numbers of branch points per field (*t*-test, SEM, *p = 0.034), of BrdU positive cells per field (*t*-test, SEM, *p ≤ 0.001), and of sprouts per 1,000 μm vessel length (*t*-test, SEM, *p ≤ 0.001) is shown. (**E**) Isolectin B4 staining (IB4, green) and BrdU labeling (red) of retinae from pups (d6) treated with solvent (co, *n* = 8) or roscovitine (rosco, *n* = 7) is shown. Scale bars (upper panels) 100 μm. Scale bars (lower panels) 50 μm. Quantifications of the area covered by ECs (*t*-test, *p ≤ 0.001), the numbers of branch points per field (*t*-test, SEM, *p = 0.005), of BrdU positive cells per field (*t*-test, SEM, *p = 0.049), and of sprouts per 1,000 μm vessel length (*t*-test, SEM, *p ≤ 0.02) is shown.

As also shown in our former study [33], Cdk5 is ubiquitously expressed in the endothelium (Figure 1A). Specific disruption of Cdk5 in the mouse endothelium using the Cre/loxP system [33] changed blood vessel patterning during development, whereas, as we could show previously, blood vessel morphology was not affected [33]. In detail, constitutive knockdown of endothelial Cdk5 with the Tie2Cre promoter [33] induced hypervascularization of yolk sacs and skin of Cdk5^fl/fl^Tie2Cre embryos (Figure 1B, 1C). Consistent with these effects, postnatal knockdown of endothelial Cdk5 with a tamoxifen-inducible VE-Cadherin Cre promoter (Cdh5(PAC)-CreERT2, *i.e.* VECCre [33, 34]) (Supplementary Figure 1A) resulted in hypervascularization of the developing retina (Figure 1D). Moreover, hypervascularization of retinae of pups treated with the small molecule Cdk5 inhibitor roscovitine demonstrated pharmacological accessibility of Cdk5 (Figure 1E). In sum, phenotyping of endothelial specific knockout mouse models revealed an important role of Cdk5 in blood vessel development.

### Endothelial knockdown of Cdk5 reduces tumor growth by promoting non-productive angiogenesis

**Figure 2:**
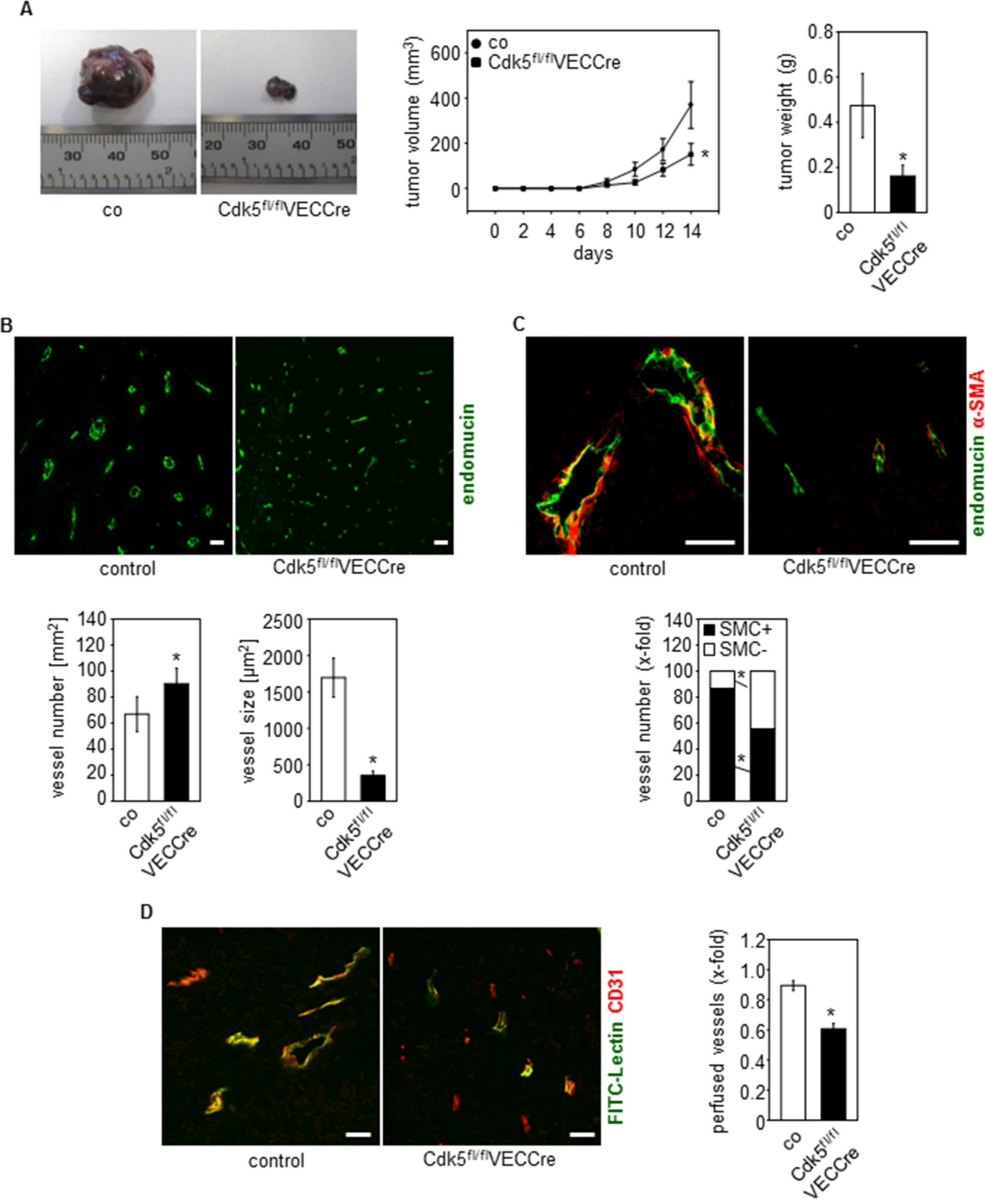
Endothelial Cdk5 knockdown reduces tumor growth with hypervascularization of tumors with non-functional vessels (**A**) Tumor growth is reduced in EC-specific Cdk5 knockout mice. B16F1 tumors from control (co, *n* = 12) and Cdk5^fl/fl^VECCre (*n* = 11) mice are shown. Time course of tumor growth (Rank Sum test, SEM, *p = 0.029) and quantification of tumor weight (Rank Sum test, SEM, *p = 0.036) is displayed. (**B**) Tumors from EC-specific Cdk5 knockout mice show hypervascularization with small blood vessels. Staining of tumors from control littermates (co, *n* = 12) and Cdk5^fl/fl^VECCre mice (*n* = 11) for endomucin (green) is shown. Scale bar 50 μm. Quantification of vessel number (Rank sum test, SEM, *p = 0.01) and vessel size (Rank sum test, SEM, *p = 0.01) is shown. (**C**) Immature tumor blood vessels in EC-specific Cdk5 knockout mice. Staining of tumors from control littermates (co, *n* = 12) and Cdk5^fl/fl^VECCre mice (*n* = 11) for endomucin (green) and α-SMA (red) is shown. Scale bar 50 μm. Quantification of the number of tumor vessels covered with SMCs (*t*-test, SEM, *p ≤ 0.002) is shown. (**D**) Tumor vessel perfusion is impaired in EC-specific Cdk5 knockout mice. FITC-lectin (green) labels perfused vessels, CD31 (red) marks blood vessels. Colocalization of FITC-Lectin green and CD31 indicates perfused vessels. Control mice (co, *n* = 5) and Cdk5^fl/fl^VECCre mice (*n* = 4). Scale bar 100 μm. Quantification of perfused tumor vessels is displayed (*t*-test, SEM, *p ≤ 0.001).

To examine the influence of endothelial Cdk5 on tumor growth, a syngeneic tumor model was applied. Tumor growth of subcutaneously implanted B16F1 melanoma cells was reduced in Cdk5^fl/fl^VECCre mice (Figure 2A and Supplementary Figure 1B). Analysis of tumor angiogenesis revealed that the number of vessels was increased in tumors of Cdk5^fl/fl^VECCre mice (Figure 2B). Interestingly, tumor vessels from Cdk5^fl/fl^VECCre mice were smaller in comparison to tumor vessels from control littermates (Figure 2B). Moreover, reduced smooth muscle cell (SMC) coverage of vessels from Cdk5 knockdown tumors demonstrated an increased incidence of immature vessels (Figure 2C). Finally, the functionality of tumor vessels was assessed by visualizing the ability of tumor vessels to perfuse FITC-lectin. Whereas control mice tumors displayed predominant overlap of FITC-lectin and CD31 staining, tumor vessels from Cdk5^fl/fl^VECCre mice were much less perfused (Figure 2D). This set of data indicates that the deletion of endothelial Cdk5 promotes non-productive angiogenesis, which resulted in reduced tumor growth.

### Cdk5 regulates the Notch pathway in the endothelium

**Figure 3:**
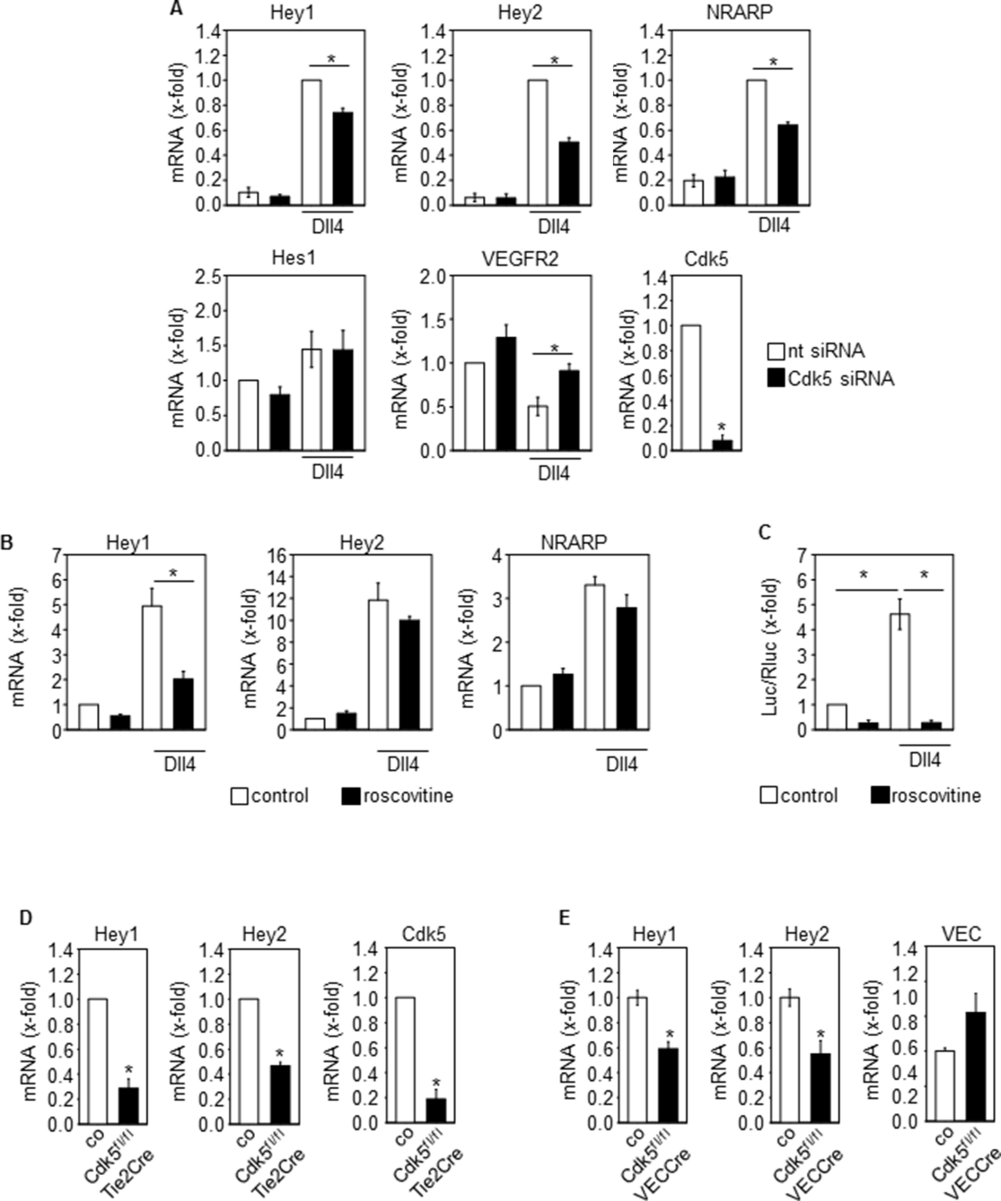
Cdk5 regulates the Notch pathway in the endothelium (**A**) Effect of Cdk5 siRNA on Dll4-induced expression of Notch downstream target genes Hey1, Hey2 and NRARP, Hes1, and VEGFR2 in HUVECs is shown (One-Way ANOVA, Holm-Sidak, SEM, *p ≤ 0.05). Cdk5 downregulation is shown (*t*-test, SEM, *p ≤ 0.001). *n* = 3. nt: non-targeting siRNA. (**B**) Effect of Cdk5 inhibition by roscovitine on Dll4-induced expression of Notch downstream target genes Hey1, Hey2 and NRARP is shown (One-Way ANOVA, Tukey, SEM, *p ≤ 0.05). *n* = 6. (**C**) Effect of Cdk5 inhibition by roscovitine on Notch reporter gene activation is shown (ANOVA on Ranks, Student-Newman-Keuls, SEM, *p ≤ 0.05). *n* = 3. (**D**) Expression of the Notch downstream target genes Hey1 and Hey2 in blood vessel endothelial cells (BECs) from E16.5 control and Cdk5^fl/fl^Tie2Cre embryos is shown (*t*-test, SEM, *p ≤ 0.001). Expression of Notch target genes was induced by plating of BECs onto Dll4. Cdk5 downregulation is shown (*t*-test, SEM, *p ≤ 0.001). *n* = 3. (**E**) Expression of the Notch downstream target genes Hey1 (*t*-test, SEM, *p = 0.003) and Hey2 (*t*-test, *p = 0.012) in tumors from control versus Cdk5^fl/fl^VECCre mice is shown. Expression of VE-Cadherin (VEC) is shown. Hey1 and Hey2 mRNA levels were normalized to VEC to compensate for the hypervascularization. *n* = 4.

**Figure 4:**
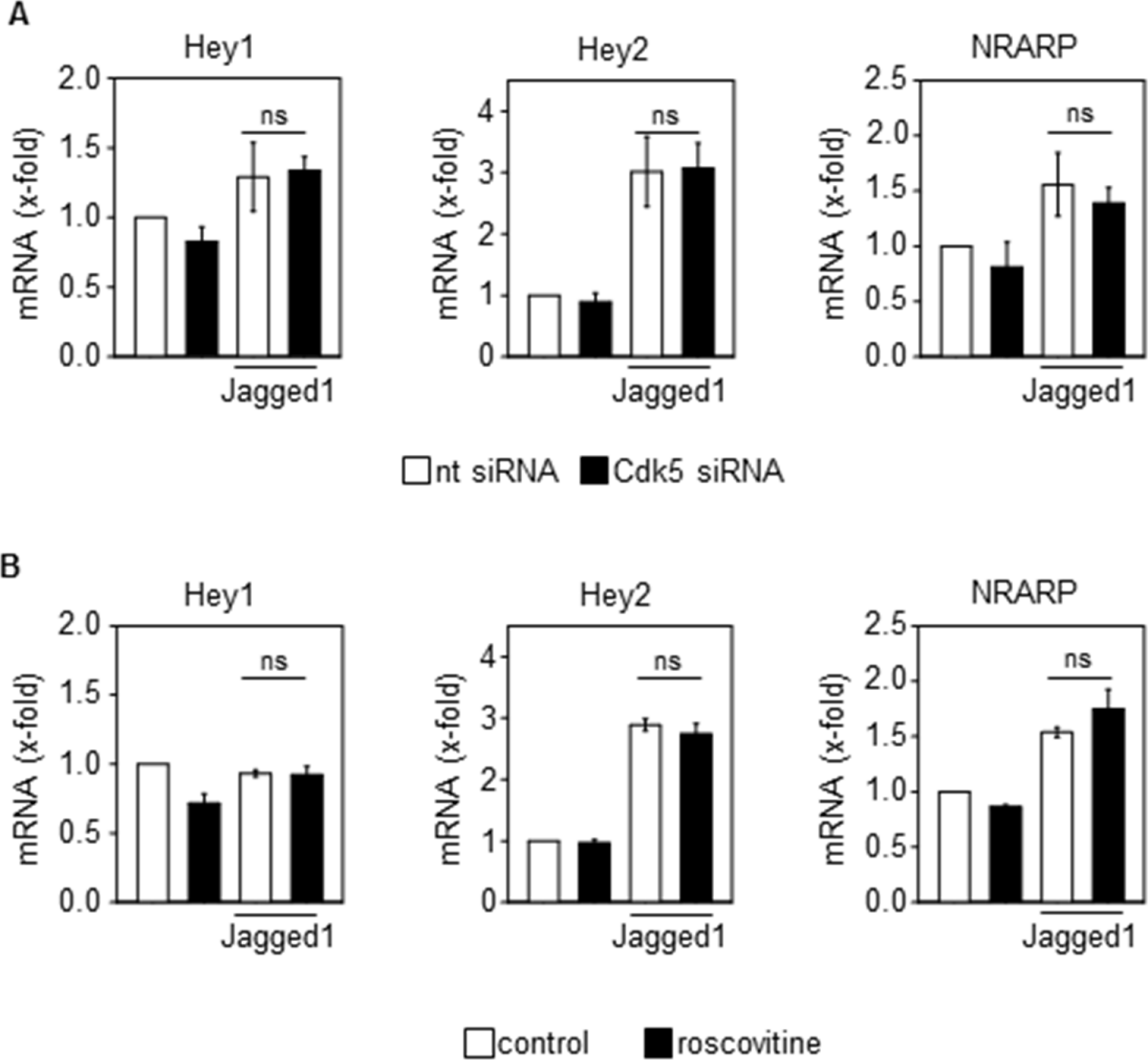
Cdk5 does not regulate the Jagged1-induced activation of the Notch pathway (**A**) Effect of Cdk5 siRNA on Jagged1-induced expression of Notch downstream target genes Hey1, Hey2 and NRARP in HUVECs is shown (One-Way ANOVA, Tukey, SEM, ns: not significant). *n* = 3. nt: non-targeting siRNA. (**B**) Effect of Cdk5 inhibition by roscovitine (10 μM, 48 h) on Jagged1-induced expression of Notch downstream target genes Hey1, Hey2 and NRARP is shown (One-Way ANOVA, Tukey, SEM, ns: not significant). *n* = 3.

Although various targets of Cdk5 have been described in cancer and endothelial cells, as one mechanism by which Cdk5 might regulate tumor angiogenesis, the Notch pathway particularly attracted our attention since the phenotype of endothelial Cdk5 knockout mice resembles that of mice with defective Dll4/Notch signaling [3]. In fact, genetic or pharmacologic inhibition of Cdk5 in endothelial cells (HUVECs) impaired Dll4-induced Notch downstream target expression (Figure 3A, 3B) as well as Notch reporter activity (Figure 3C). Consistent with these effects, blood vessel endothelial cells (BECs) from Cdk5^fl/fl^Tie2Cre embryos displayed reduced expression of Notch target genes after Notch activation by Dll4 (Figure 3D). Furthermore, the expression of the Notch downstream target genes Hey1 and Hey2 was decreased in tumors from endothelial Cdk5 knockout mice (Figure 3E). All these findings point to a Cdk5-Notch pathway for the regulation of angiogenesis. However, genetic or pharmacological inhibition of Cdk5 did not abrogate the Jagged1-induced Notch target expression (Figure 4A, 4B), suggesting a specific regulation of Dll4-driven Notch signaling by Cdk5.

### Cdk5 regulates NICD generation

**Figure 5:**
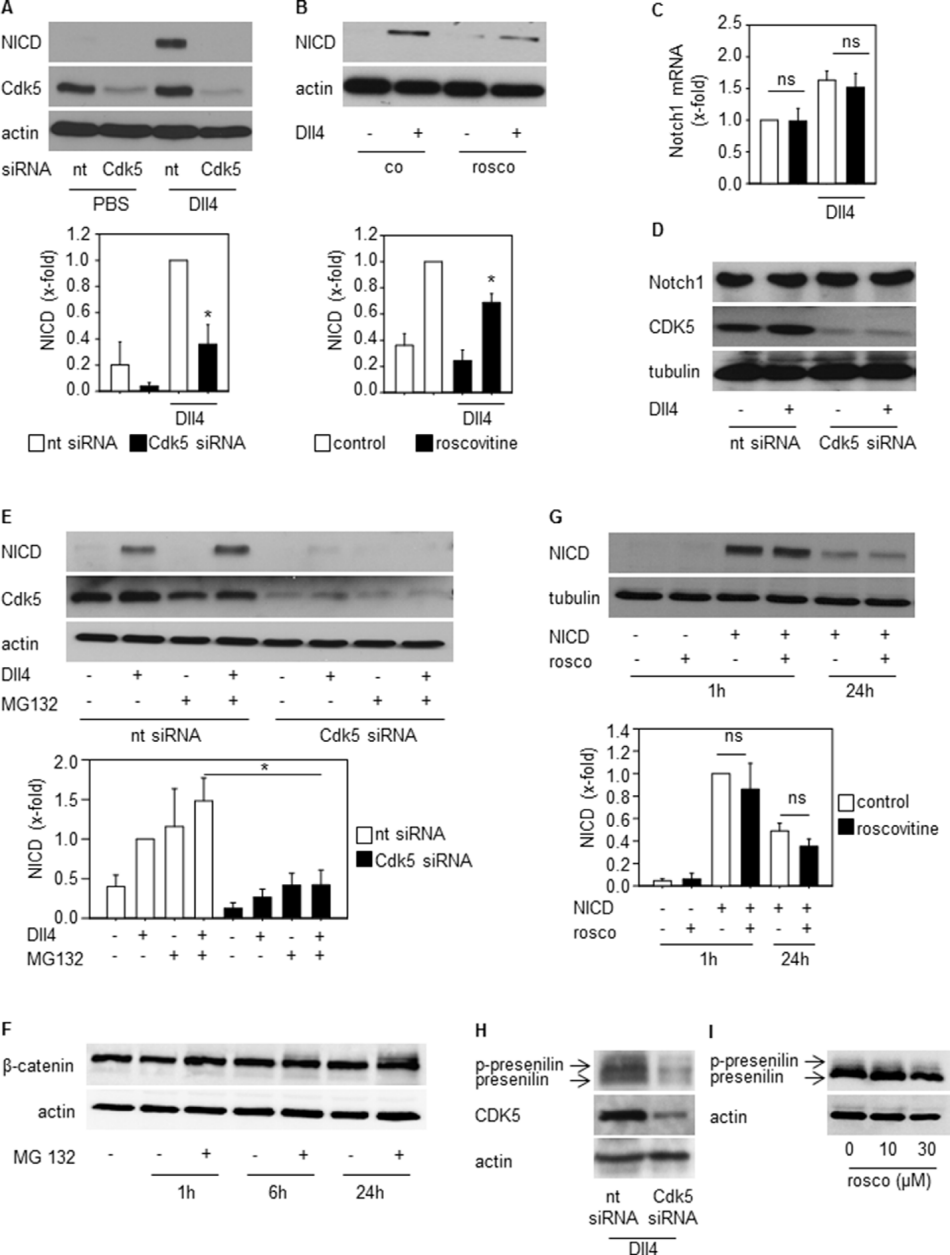
Cdk5 regulates NICD generation (**A**) Immunoblots from HUVECs transfected with nt (non-targeting) and Cdk5 siRNA and plated onto PBS- or DLL4-coated dishes probed with anti-NICD, anti-Cdk5, and anti-actin antibodies are shown. *n* = 3. The bar graph displays the quantification of the NICD immunoblot normed to the loading control (One Way ANOVA, Tukey, SEM, *p ≤ 0.05). (**B**) Immunoblots from HUVECs untreated (co) or treated with roscovitine (rosco, 10 μM), plated onto PBS- or Dll4-coated dishes and probed with anti-NICD and anti-actin antibodies are shown. *n* = 3. The bar graph displays the quantification of the NICD immunoblot normed to the loading control (One Way ANOVA, Tukey, SEM, *p ≤ 0.05). (**C**) Expression of Notch1 receptor mRNA of HUVECs transfected with nt (non-targeting) or Cdk5 siRNA is shown. One-Way ANOVA, Tukey, *n* = 3. (**D**) Immunoblots from HUVECs transfected with nt (non-targeting) or Cdk5 siRNA and probed with anti-Notch, anti-Cdk5, and anti-tubulin antibodies are shown. *n* = 3. (**E**) Immunoblots of HUVECs silenced with nt (non-targeting) or Cdk5 siRNA and treated with MG132 for 24 h before plating onto Dll4 and probed with anti-NICD, anti-Cdk5 and anti-actin antibodies are shown. *n* = 3. The bar graph displays the quantification of the NICD immunoblot normed to the loading control (One Way ANOVA, Tukey, SEM, *p ≤ 0.05). (**F**) Immunoblots of HUVECs treated with MG132 for the indicated timepoints and probed with anti-β-catenin and anti-actin antibodies are shown. *n* = 2. The bar graph displays the quantification of the NICD immunoblot normed to the loading control (One Way ANOVA, Tukey, SEM, ns: not significant). (**G**) Immunoblots of HUVECs overexpressing NICD or empty vector treated with/without roscovitine for the indicated times and probed with anti-NICD and anti-tubulin antibodies are shown. *n* = 3. (**H**) Immunoblots of HUVECs transfected with nt (non-targeting) or Cdk5 siRNA plated onto Dll4 and probed with anti-presenilin, anti-Cdk5, and anti-actin antibodies are shown. Phosphorylated and total presenilin is denoted by the upper and lower band and marked by arrows. *n* = 3. (**I**) Immunoblots of HUVECs treated with/without roscovitine at indicated concentrations plated onto Dll4 and probed with anti-presenilin and anti-actin antibodies are shown. Phosphorylated and total presenilin is denoted by the upper and lower band and marked by arrows. *n* = 3.

To understand the link between Cdk5 and the Dll4-Notch pathway, further experiments focused on the regulation of the Notch intracellular domain (NICD), the key mediator of Notch signaling [35]. After ligand-induced proteolytic cleavage of the Notch receptor, NICD is released and translocates to the nucleus to drive target gene transcription before the signal is terminated by proteasomal degradation of NICD [35]. Inhibition of Cdk5 by either knockdown or pharmacologic approaches decreased Dll4-induced NICD generation (Figure 5A, 5B). This was not based on changed Notch receptor expression as Cdk5 silencing neither influenced Notch receptor mRNA (Figure 5C) nor protein (Figure 5D). Furthermore, the inhibition of the proteasomal degradation by MG132 did not abrogate the Cdk5 siRNA mediated decrease of NICD (Figure 5E). To prove the functionality of MG132, β-catenin was used as a positive control as it is degraded by the proteasome [36]. Increased β-catenin in the presence of MG132 proved that MG132 inhibited the proteasomal degradation (Figure 5F). In line, exogenously expressed NICD that is not dependent on Notch receptor cleavage, was not affected by Cdk5 inhibition (Figure 5G). Both results suggest that Cdk5 preferentially contributes to NICD generation rather than its degradation. Cdk5 knockdown by siRNA as well as Cdk5 inhibition by roscovitine decreased levels of phosphorylated and total presenilin, the catalytic subunit of the γ-secretase multiprotein complex capable of mediating NICD generation. (Figure 5H, 5I). This set of data suggests that Cdk5 provides negative tonus on Notch-dependent signaling.

### Cdk5 regulates Notch dependent endothelial functions

**Figure 6:**
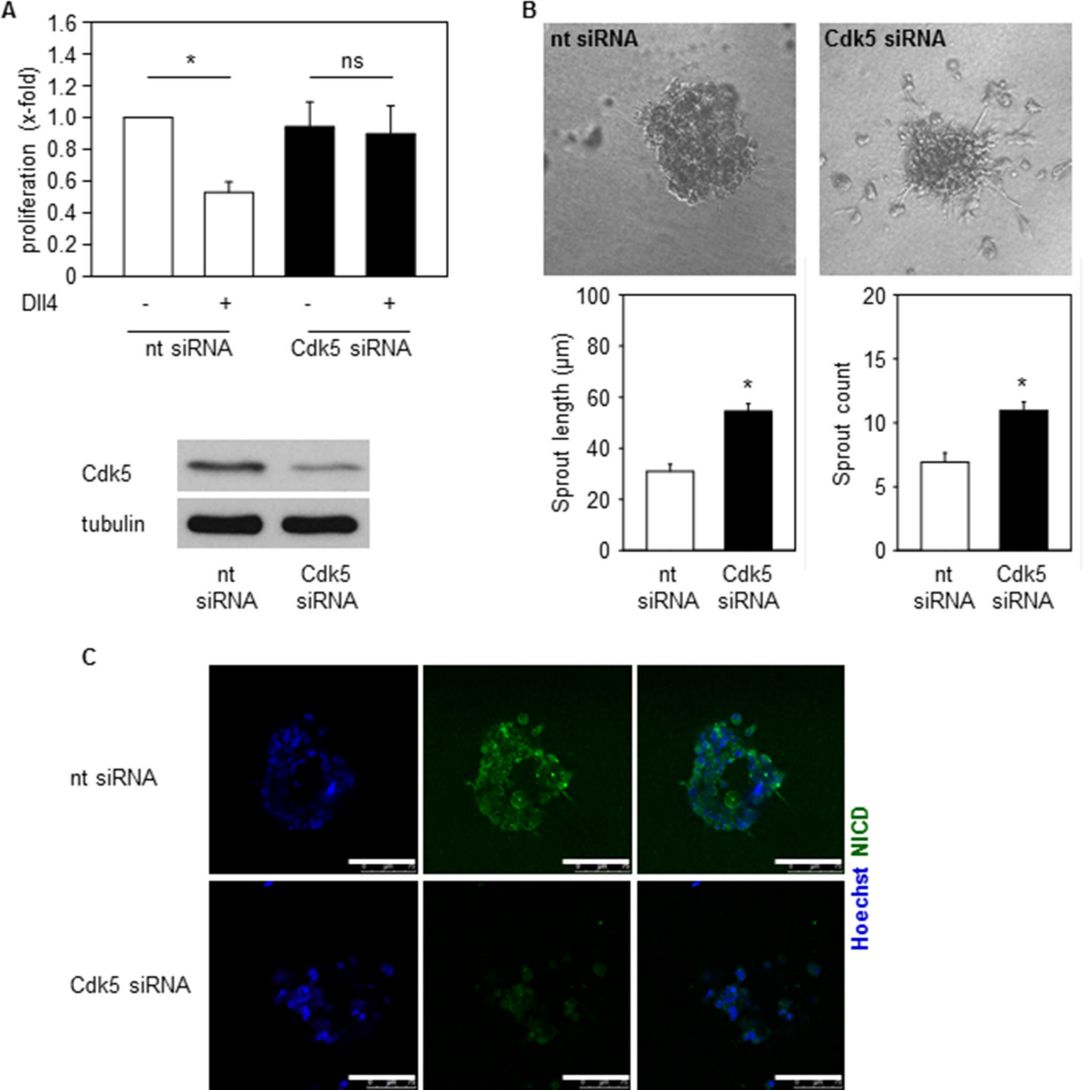
Cdk5 regulates Notch pathway dependent endothelial functions (**A**) Proliferation of HUVECs transfected with nt (non-targeting) or Cdk5 siRNA and plated onto PBS or Dll4 is shown. One-Way ANOVA, Tukey, SEM *p ≤ 0.05. *n* = 4. (**B**) Spheroids generated from HUVECs transfected with nt (non-targeting) or Cdk5 siRNA embedded into Dll4 containing gels are shown. Quantifications of sprout length and number of sprouts are displayed. *T*-test, SEM, *p ≤ 0.001. Number of evaluated spheroids: non-targeting siRNA *n* = 21, Cdk5 siRNA *n* = 26. (**C**) Immunostainings of spheroids generated from nt (non-targeting) or Cdk5 siRNA transfected HUVECs for NICD (green) and Hoechst 33342 (blue) are shown. Scale bar 100 μm. *n* = 3.

We next asked if Cdk5 could contribute to Notch dependent endothelial functions like proliferation and sprouting of endothelial cells [37, 38]. In fact, inhibition of endothelial Cdk5 abrogated the Dll4-mediated decrease of endothelial cell proliferation (Figure 6A). In line, Cdk5 inhibition increased sprouting of spheroids embedded into Dll4-containing gels (Figure 6B). Moreover, in line with the immunoblots in Figure 5 that showed reduced NICD levels by Cdk5 inhibition, immunostainings suggest reduced NICD levels in spheroids generated from Cdk5 siRNA treated cells (Figure 6C). Thus, inhibition of Cdk5 impaired endothelial cell functions which are dependent on Dll4-driven Notch activation.

### Cdk5 inhibition reduces tumor growth and improves sensitivity to anti-angiogenic treatment

**Figure 7:**
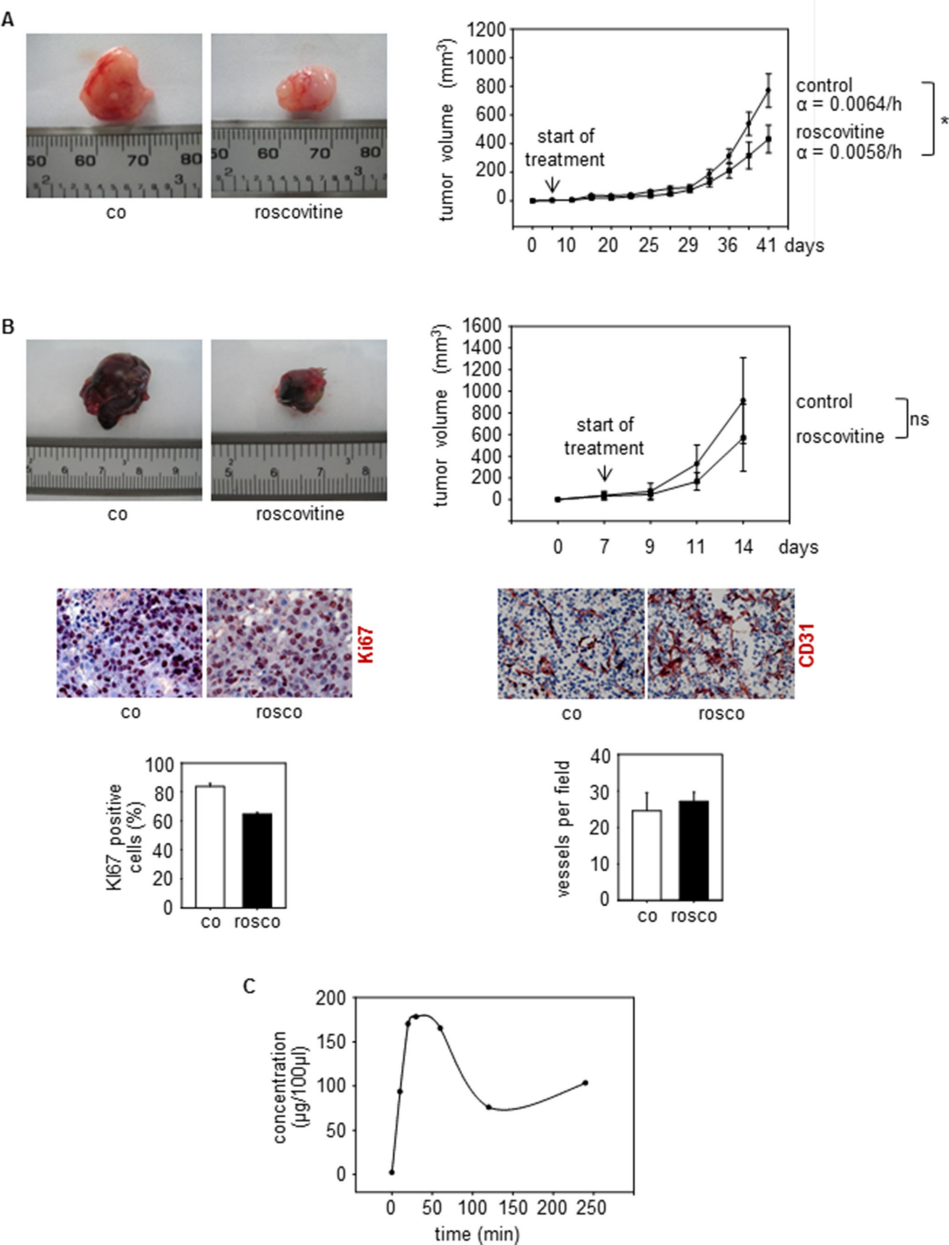
Cdk5 inhibition reduces tumor growth (**A**) U87 tumors from mice treated with solvent (co) or roscovitine (rosco) is shown. *n* = 5. The graph shows tumor growth over time. Growth rate α of tumors is indicated (*p = 3 × 10^−7^). (**B**) B16F1 tumors from Cdk5^fl/fl^VECCre mice treated with solvent (co) or roscovitine (rosco) is shown. The bar graph shows tumor growth over time (*t*-test, ns: not significant, *n* = 3). Staining of tumors Cdk5^fl/fl^VECCre mice for Ki67 (red, upper panel) and CD31 (red, lower panel) is shown (*n* = 2). The bar graphs display respective quantifications. (**C**) Pharmakokinetics of Roscovitine in mice is shown. The graph displays the concentration (μg per 100 μl) of Roscovitine in blood of mice after i.p. injection at the indicated timepoints. *n* = 3 mice per timepoint.

As described above, knockout of endothelial Cdk5 reduced tumor growth of wildtype B16F1 melanoma cells (Figure 2A), demonstrating that endothelial Cdk5 regulates tumor growth. To investigate the influence of Cdk5 inhibition on tumor growth in a more therapeutic context, we used systemic treatment with the small molecule Cdk5 inhibitor roscovitine. Although, like most kinase inhibitors, roscovitine is not selective for the inhibition of Cdk5 but also addresses other Cdks like Cdk1, Cdk2, Cdk7, and Cdk9, it represents the best-established inhibitor for Cdk5 and therefore was used as a model substance for our studies [39–41]. We used a subcutaneous human U87 glioblastoma cell xenograft model as glioblastoma represent highly vascularized and aggressive tumors [42]. Roscovitine was given after tumors had established and reduced glioblastoma growth as shown by a significantly reduced growth rate of tumors from roscovitine treated mice (Figure 7A). In endothelial-specific Cdk5 knockout mice, systemic treatment with roscovitine slightly but not significantly reduced tumor growth and decreased tumor cell proliferation as shown by Ki67 staining (Figure 7B). Moreover, roscovitine had no effect on vessel number in tumors grown in endothelial Cdk5 knockout mice (Figure 7B). This set of data suggests that Cdk5 indeed is the primary target of roscovitine, but that roscovitine also acts on tumor cells as well and inhibits other Cdks besides Cdk5. Pharmacokinetic studies showed plasma concentrations of roscovitine comparable to doses used in our cell-based assays (Figure 7C).

**Figure 8:**
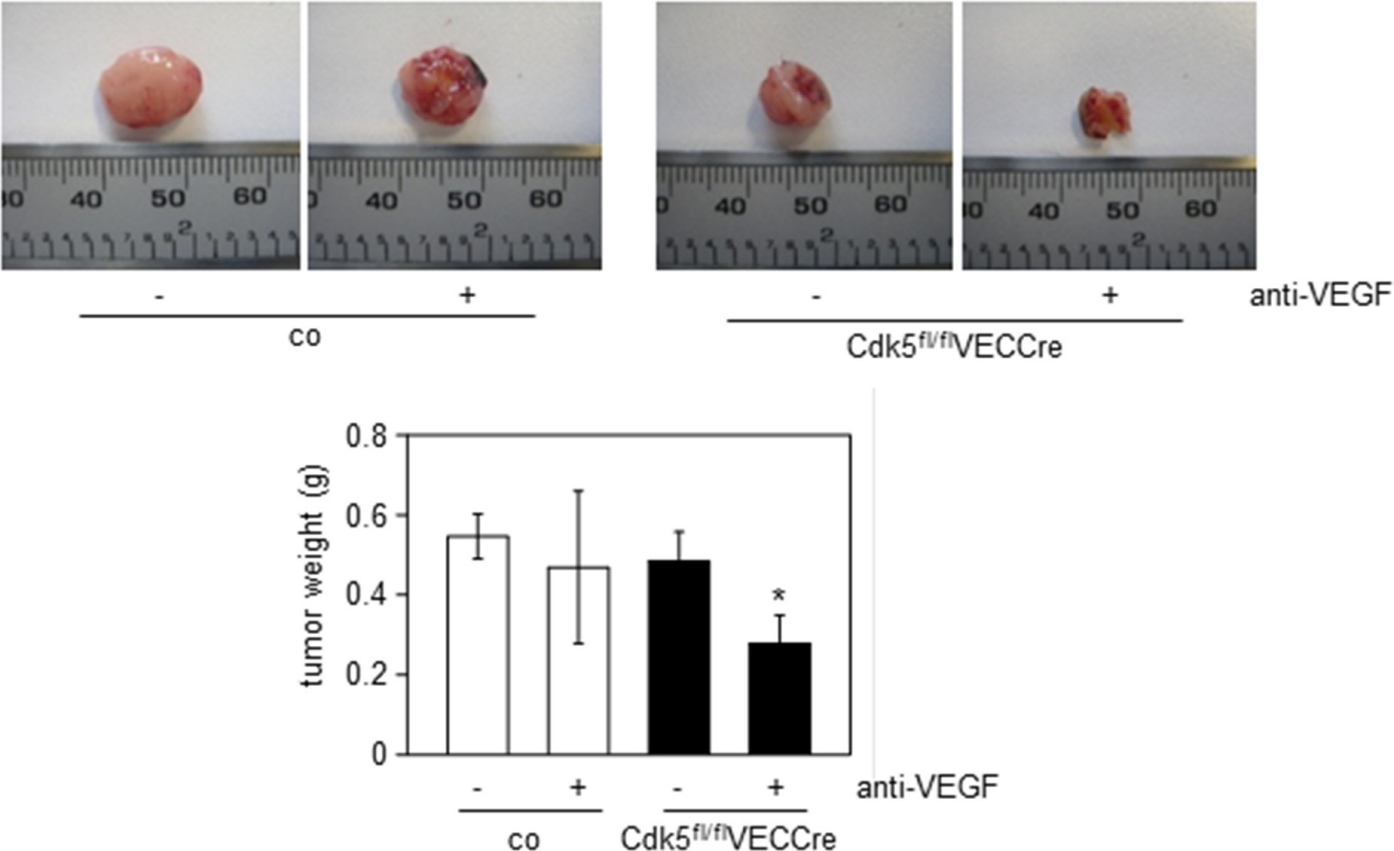
Cdk5 inhibition sensitizes to anti-angiogenic treatment LLC tumors from control (co) or Cdk5^fl/fl^VECCre mice treated with solvent or the anti-VEGF antibody B20–4.1.1 are shown. Quantification of tumor weight is indicated. ANOVA on Ranks, Dunn's Method, SEM, *p ≤ 0.05. control/solvent *n* = 10, control/anti-VEGF *n* = 9, Cdk5^fl/fl^VECCre/solvent *n* = 9, Cdk5^fl/fl^VECCre/anti-VEGF *n* = 8.

Together with the reduced growth of wildtype tumors in endothelial Cdk5 knockout mice (Figure 2A), this set of data provides evidence for Cdk5 as a drugable target for anti-angiogenic therapy. Finally, we assessed the effect of anti-VEGF treatment on Lewis lung carcinoma (LLC) growth in endothelial-specific Cdk5 knockout mice. The LLC model has been described to be resistant to conventional anti-angiogenic therapy [43]. As expected, the anti-VEGF antibody B20–4.1.1 did not reduce LLC tumor growth in control mice. However, in endothelial Cdk5 knockout mice anti-VEGF treatment diminished tumor growth (Figure 8). Thus, Cdk5 inhibition enhanced sensitivity of tumors to anti-VEGF treatment.

## DISCUSSION

Substantial advances in understanding of angiogenesis have been made with regard to the regulation and targeting of growth factor receptors such as the VEGF-Receptor [1, 44, 45]. However, the inhibition of growth factors is not effective in all cancers [46]. Therefore, it is essential to further understand how the tumor vasculature can be effectively targeted in order to develop new anti-angiogenic therapies. However, the knowledge about intracellular processes that mediate neovascularization is still limited.

Here, we show that endothelial Cdk5 contributes to blood vessel development and tumor angiogenesis. In doing so, this work provides the first evidence for an *in vivo* role of Cdk5 in blood vessel formation. There have been various reports including ours, describing functions of Cdk5 in endothelial cells: in detail, endothelial Cdk5 was implicated in endothelial senescence [47], in neovascularization after ischemic stroke [48], and in diseases associated with NO dysfunction [49, 50]. Our former studies demonstrated that small molecule Cdk5 inhibitors exert anti-angiogenic effects and that Cdk5 is implicated in endothelial cell migration by regulating the small GTPase Rac1 which was restricted to *in vitro* assays. However, the specific *in vivo* role of Cdk5 in the blood vessel endothelium by genetic knockdown models has not been addressed to date. Of note, as we started to analyze the *in vivo* function of Cdk5 in the endothelium, we observed no changes in arterial-venous cell specification and blood vessel morphology by Cdk5 knockdown in the endothelium [33]. In fact, we found that Cdk5 is essential for lymphatic vessel development and valve formation by phosphorylating the transcription factor Foxc2 [33]. Our present study though elucidates a crucial role of Cdk5 in blood vessel patterning.

With respect to the mechanisms and potential targets of Cdk5 action in blood vessels, rather the Dll4/Notch system was indicated to be important, as suggested by the reminiscent phenotype of Cdk5 knockout mice to angiogenesis observed by Dll4/Notch blockade. Similar to the genetic or pharmacologic inhibition of endothelial Cdk5, the disruption of Dll4/Notch signaling results in increased vessel sprouting during development [3]. Dll4/Notch signaling regulates tip and stalk cell specification during vascular morphogenesis. Dll4 is induced in endothelial tip cells and activates Notch in adjacent stalk cells which represses the tip cell phenotype. Inhibition of the Notch pathway results in increased vessel sprouting and branching due to excessive tip cell formation and endothelial cell proliferation. Moreover, in line with our results showing dysfunction of tumor vessels in endothelial Cdk5 knockout mice, preclinical models have demonstrated that blockade of Dll4/Notch signaling results in increased vessel sprouting and branching in tumors and impairs tumor growth by promoting non-productive angiogenesis [6, 7]. With respect to the target of Cdk5 within the Dll4/Notch signaling hub our work points to an alteration of presenilin/γ-secretase and therefore the regulation of NICD generation by endothelial Cdk5. In neurons presenilin/γ-secretase was identified as Cdk5 target before [51]. Presenilin comprises the catalytic component of the γ-secretase multiprotein complex that is essential for Notch receptor processing and NICD generation [35].

Therapeutic targeting of the Dll4/Notch pathway has evolved as an attractive anti-angiogenic strategy as the adaptive ability of neoplastic cells to become anti-VEGF resistant has arisen as a major obstacle to anti-angiogenic therapies [2] and can be caused by compensation of alternative angiogenesis mechanisms such as the Dll4/Notch pathway [5, 8, 9]. Potentiated Notch signaling due to either loss of a negative regulator or increased expression of the Notch activating ligand Dll4 in the tumor vasculature correlates with tumorigenesis and therapeutic resistance [4, 5, 10, 52, 53]. In breast cancer, high Dll4 expression by intratumoral endothelial cells was elucidated as an adverse prognostic factor of patient survival [54]. In line, strong expression of Dll4 in ovarian cancer was associated with poor patient prognosis whereas low Dll4 expression correlated with responsiveness to anti-VEGF therapy [8]. Recently, it was shown that the modulation of Dll4/Notch by the extracellular matrix (ECM) protein fibulin-3 promotes angiogenesis in high-grade gliomas [42]. However, whereas Dll4 expression in tumors consistently affected the vascular phenotype, tumor growth was increased only in some tumors, probably due to differences in the vasculature and/or levels of endogenous components of the Dll4/Notch pathway [4, 6]. In line, our study shows that Cdk5 inhibition alone inhibited B16F1 melanoma growth but not LLC tumor growth. Importantly, Dll4/Notch blockade has been shown to inhibit the growth of anti-VEGF resistant tumors and even enhance the sensitivity of tumors to anti-VEGF treatment [5, 8, 9]. Consequently, various approaches have been developed to target the Dll4/Notch pathway including anti-Dll4 antibodies, Dll4-Fc and Notch-Fc decoys, DNA vaccination, anti-Notch antibodies, as well as γ-secretase inhibitors and anti-Dll4/Notch therapy is currently evaluated in clinical trials for cancer therapy [9]. Still, the molecular basis of this sensitizing effect has not been well explained [9]. Here we show that Cdk5 inhibition within the neovascular endothelium of burgeoning tumors can sensitize them for more effective anti-angiogenic treatment. This may be of particular relevance to tumors such as lewis lung carcinoma (LLC) which have been described to be resistant to anti-angiogenic treatment [43]. Thus, the control of Notch signaling by Cdk5 in the neovascularizing endothelium is proposed as an additional target for tumor treatment although up to now, absolutely selective inhibition specifically of Cdk5 is not possible as the currently available Cdk5 inhibitors interfere with other Cdks like Cdk1, Cdk2, Cdk7, and Cdk9 as well. Nevertheless, inhibition by small molecules inhibiting Cdk5 and additionally Cdk1, Cdk2, Cdk7, and Cdk9 have shown promising effects in cancer/angiogenesis [18, 30, 31, 55, 56]. In fact, as inhibition of several kinases can address different functions in endothelial as well as in tumor cells, and therefore interfere with tumor growth and progression in a multifaceted mode of action, this might be beneficial in terms of therapeutic efficiency.

In sum, the present study elucidates an essential *in vivo* role of Cdk5 in tumor angiogenesis suggesting Cdk5 inhibition as a novel approach for anti-angiogenic treatment.

## MATERIALS AND METHODS

### Animal experiments

All animal experiments were performed with approval by the District Government of Upper Bavaria in accordance with the German animal welfare and institutional guidelines.

### Endothelial-specific Cdk5 knockout mice

Generation, breeding, and genotyping of endothelial-specific Cdk5 knockout mice was previously described [33]. Floxed Cdk5 mice were described [57]. Tie2Cre mice were from Jackson Laboratory (B6.Cg-Tg(Tek-cre)12Flv/J, 004128). Tamoxifen-inducible Cdh5(PAC)-CreERT2 mice were described [34]. Endothelial Cdk5 knockdown in pups was achieved by tamoxifen injection at day 1 – day 3 (50 μg/day i.p., Sigma Aldrich). Cdk5 knockdown was proved at day 6 (Supplementary Figure 1A). For deletion of Cdk5 in adult mice, tamoxifen (0.5 mg/day i.p.) was injected at 5 consecutive days. Cdk5 knockdown was proved two weeks and four weeks after treatment with tamoxifen (Supplementary Figure 1B).

### Tumor models

B16F1 melanoma cells (1 × 10^6^ cells in 100 μl PBS) were subcutaneously injected into the flanks of 8 week old Cdk5^fl/fl^VECCre and control mice at day 15 after tamoxifen injection when Cdk5 was downregulated. Tumor growth was observed for 14 days (until d30). Tumor volume was evaluated every 2nd day (π/6 × l × w × h). Tumor weight was evaluated. In case of therapy with roscovitine, mice were intraperitoneally treated with roscovitine (150 mg/kg, 3 × per week) starting from day 7 after tumor cell injection, when tumors had established. The tumor volume was evaluated three times per week. Mice were sacrificed at day 14 and the tumor weight was determined.

U87 glioblastoma cells [4, 5] were subcutaneously injected (5 × 10^6^ cells in 100 μl PBS:Matrigel 1:1) into the flanks of 6 week old female Balb/c nude mice (Harlan). Mice were treated intraperitoneally (i.p.) with roscovitine (150 mg/kg, 3 × per week) starting at day 7 after tumor cell implantation when tumors had established and the tumor volume was evaluated twice per week. Mice were sacrificed at day 42. Tumor volume was modelled using an exponential growth model where the tumor volume at a given time t (N(t)) is a function of the starting volume N (0), the time of growth *t* and of a growth rate α: N (t) = N (0) • exp^α • t^. Modeling was performed using non-linear mixed effects modeling with the software NONMEM 7.3. [58].

LLC cells (2 × 10^6^ cells in 100 μl PBS) cells were subcutaneously injected into the flanks of 8 week old Cdk5^fl/fl^VECCre and control mice at day 15 after tamoxifen injection when Cdk5 was downregulated. Starting at d3 after tumor cell implantation, mice were treated with anti-VEGF antibody (B20–4.1.1, Genentech, 5 mg/kg 2 ×/week, i.p.) or solvent (PBS) respectively. Mice were sacrificed at day 18 and tumor weight was evaluated.

### Pharmacokinetics

Plasma concentrations of roscovitine were determined by HPLC-DAD. Mice were treated with roscovitine and blood was collected after 10, 20, 30, 60, 120, and 240 min. For each time point, blood of three mice was pooled.

100 μl mice blood were mixed with 200 μl acetonitrile, vortexed (1 min), centrifuged (5 min, 10,000 g, RT) and the supernatant was analyzed by HPLC-DAD using an Agilent Series 1100 HPLC system (Waldbronn, Germany) consisting of a quaternary pump system (G1311 A QuatPump), an autosampler (G1329 A ALS), a column oven (G1316 A ColComp) and a UV-DAD detector (G1315 A DAD). Chromatographic separation was carried out with an Agilent poroshell 120 EC-C18 (100 × 3.0 mm, i.d. 2.7 μm) column (Waldbronn, Germany) and a mobile phase of acetonitrile and water (0.1% phosphoric acid, 1.0% tetrahydrofuran) 15:85 (v/v). The total run time was 7 min with an isocratic flow rate at 1.0 ml/min, and an injection volume of 10 μl. The column oven was set at 50°C. The UV detection wave-length was set at 292 nm. Data analysis and instrument control was carried out with Agilent ChemStation^®^ software Rev. B04.02. The average retention time of roscovitine was 3.1 min. The concentration of roscovitine was determined according to an external standard calibration.

### Retina preparation and staining

Retina preparation and staining was performed according to Pitulescu et al. [59]. Briefly, eyes were removed, fixed in PFA 4% (2 h, RT) and retinae were prepared. After blocking (2 h, RT), retinae were stained for isolectin B4 (IB4, Alexa 488 conjugated, Millipore), and BrdU staining was performed. Nuclei were labeled with Hoechst33342. Pictures were taken with a Zeiss LSM 510 META confocal microscope. The area covered by ECs, the numbers of branch points per field, number of BrdU positive cells per field, and of sprouts per 1,000 μm vessel length were calculated by using Image J.

### Immunohistochemistry

Paraffin sections: Tumors were removed, fixed with PFA 4% for 24 h, left in PFA 1%, embedded into paraffin and sections (5 μm) were prepared.

Cryosections: Tumors were removed and frozen into TissueTek. 10 μm sections were prepared and fixed with formalin 4% (10 min, RT).

Stainings: Sections were blocked (1% BSA/PBS), incubated with primary antibodies (CD31, 553370, BD Pharmingen; endomucin, sc-65495 Santa Cruz; α-SMA C6198, Sigma) for 2 h, at RT or o/n at 4°C, washed, incubated with AlexaFluor-labeled secondary antibodies (45 min, RT, Life Technologies) and Hoechst 33342 (5 μg/ml) and mounted (Fluorsave Reagent, Calbiochem). Pictures were taken with a Zeiss LSM 510 META confocal microscope.

For evaluations of stainings, ImageJ and the particle counter plugin were used. Vessel number was determined by counting the number of vessels per mm^2^. Vessel size was determined by evaluating the area covered by vessels divided by the number of vessels per μm^2^. For the quantification of smooth muscle cell coverage of vessels, tumor sections were stained for CD31 and α-smooth muscle cell actin (α-SMA). Vessels with and without αSMA-staining were counted.

### Whole mount staining of embryonic skin and yolk sacs

Tissues were removed, fixed (formalin 4%, 30 min, RT), washed, blocked (1 h, RT, 0.5% TritonX, 2% BSA/PBS), incubated with anti-CD31 primary antibodies (553370, BD Pharmingen) (o/n, 4°C), washed, incubated with AlexaFluor-labeled secondary antibodies (2 h, RT), and mounted. Numbers of branch points per field were calculated by using Image J and the particle counter plugin.

### Tumor vessel perfusion

At day 15 after tumor cell inoculation, mice were intravenously injected (tail vein) with FITC-Lectin (150 μg, 1 mg/ml, Sigma Aldrich). After circulation of FITC-Lectin for 5 min, mice were sacrificed and tumors were removed. Staining was performed according to the description under immunohistochemistry. Vessels with and without FITC-Lectin labeling were counted by using ImageJ and the particle counter plugin.

### Cells

HUVECs were cultivated as described [32] with Endothelial Cell Growth Medium (ECGM, Promocell) containing 10% FCS. Embryonic blood vessel endothelial cells (BECs) and liver sinusoidal endothelial cells LSECs were isolated and cultivated as described [33]. For experiments with Dll4, plates were coated with Dll4 (5 μg/ml; 1 h RT or o/n 4°C; human Dll4 for HUVECs and mouse Dll4 for mouse BECs, both R & D Systems 1506-D4 and 1389-D4). Plates were washed once with PBS before cells were plated. For proteasome inhibition experiments, MG132 (1 μM, Enzo Life Sciences) stimulation was started 1 h before re-plating of HUVECs onto Dll4. HUVECs were plated onto Dll4 for 1 h or 24 h. BECs were plated onto Dll4 for 24 h.

For experiments with Jagged1, plates were coated with Jagged1 (10 μg/ml; 1 h RT; R & D Systems 1277-JG). Plates were washed once with PBS before cells were plated. HUVECs were treated with roscovitine (10 μM) or nt (non-targeting) or Cdk5 siRNA and plated onto Dll4 for 48 h.

### Transfection of cells

HUVECs were transfected using Targefect (Targeting Systems; El Cajon, California) according to the manufacturer's protocol. DNA, Transfection Media, Targefect and Virofect Enhancer were mixed and incubated for 25 min. The transfection complex was added to the cells for 2 h. Fresh HUVEC medium was added to the cells for 24 h. NICD plasmid was from addgene (26892). The siRNAs were from Thermo Scientific/Dharmacon: nt siRNA D-001810–01; Cdk5 siRNA J-003239–09 and J-003239–10. For the experiments involving Cdk5 siRNA, a mix of both siRNAs was used.

### Proliferation

HUVECs were transfected with nt or Cdk5 siRNA (Thermo Scientific). 24 h after transfection, cells (1500 cells per 96-well) were seeded onto PBS or Dll4 coated plates. Initial cell number was determined after 1 h. Proliferation was measured after 72 h via crystal violet staining.

### Spheroids

HUVECs were transfected with nt or Cdk5 siRNA. Spheroids were generated via the hanging drop method as described [37]. Briefly, a suspension of HUVECs and 20% Methocel was mixed and 20 μl drops were seeded onto a petri dish and incubated upside down over night. Spheroids were embedded into HUVEC growth medium containing 5% Minimal Essential Eagle's Medium (Sigma), 5% Bicarbonate, 60% collagen and Dll4 (5 μg/ml). The collagen pellets were covered with ECGM containing 20% FCS. Pictures were taken on day 0, 1, 2 and 3. Sprout length and number of sprouts were determined.

For spheroid staining, embedded spheroids were fixed with 4% PFA, permeabilized with 0, 2% Triton X and blocked with 1% BSA in PBS for 3 days. Primary antibody (NICD 4147, Cell Signalling) was incubated for 3 days and secondary antibody (AlexaFluor 488, Life Technologies) for further 3 days. Nuclei were stained with Hoechst 33342 (0, 5 μg/ml, 40 min, RT). Pictures were taken at the Leica SP8 SMD confocal microscope.

### Immunoblotting

Immunoblotting was described previously [33]. The following primary antibodies were used: actin (MAB 150 1R, Chemicon), Cdk5 (AHZ0492, Life Technologies), NICD (4147 Cell Signalling), presenilin (5643, Cell Signaling).

### RT-PCR

mRNA was isolated using the Qiagen RNeasy Mini Kit. For reverse transcription the High-Capacity cDNA Reverse Transcription Kit (Applied Biosystems) was used. RT-PCR was performed with the 7300 Real Time PCR System. The following Taqman gene expression assays were used: Cdk5 Hs00358991_g1 and Mm01134945_g1, Hey1 Hs00232618_m1 and Mm00468865_m1, Hey2 Hs00232622_m1 and Mm00469280_m1, NRARP Hs01104102_s1 and Mm00482529_s1, VE-cadherin Mm00486938_m1 (Applied Biosystems). GAPDH was used as housekeeper.

### Statistics

All experiments were performed at least three times (biological replicates) Respective tests, *p*-values and exact numbers of independently performed experiments are indicated in the respective figure legends. Graph data represent means ± SEM. Statistical analysis was performed using SigmaStat Version 3.1.

## SUPPLEMENTARY MATERIALS AND FIGURE


