# Supplementary material for: Inhibition of endothelial Cdk5 reduces tumor growth by promoting non-productive angiogenesis
Source: Oncotarget. 2016 Jan 8;7(5):6088–104. doi: 10.18632/oncotarget.6842 (PMC4868742; doi:10.18632/oncotarget.6842)
Supplement: Supplementary file 1 [file oncotarget-07-6088-s001.pdf]

Inhibition of endothelial Cdk5 reduces tumor growth by promoting non-productive angiogenesis

Supplementary Materials

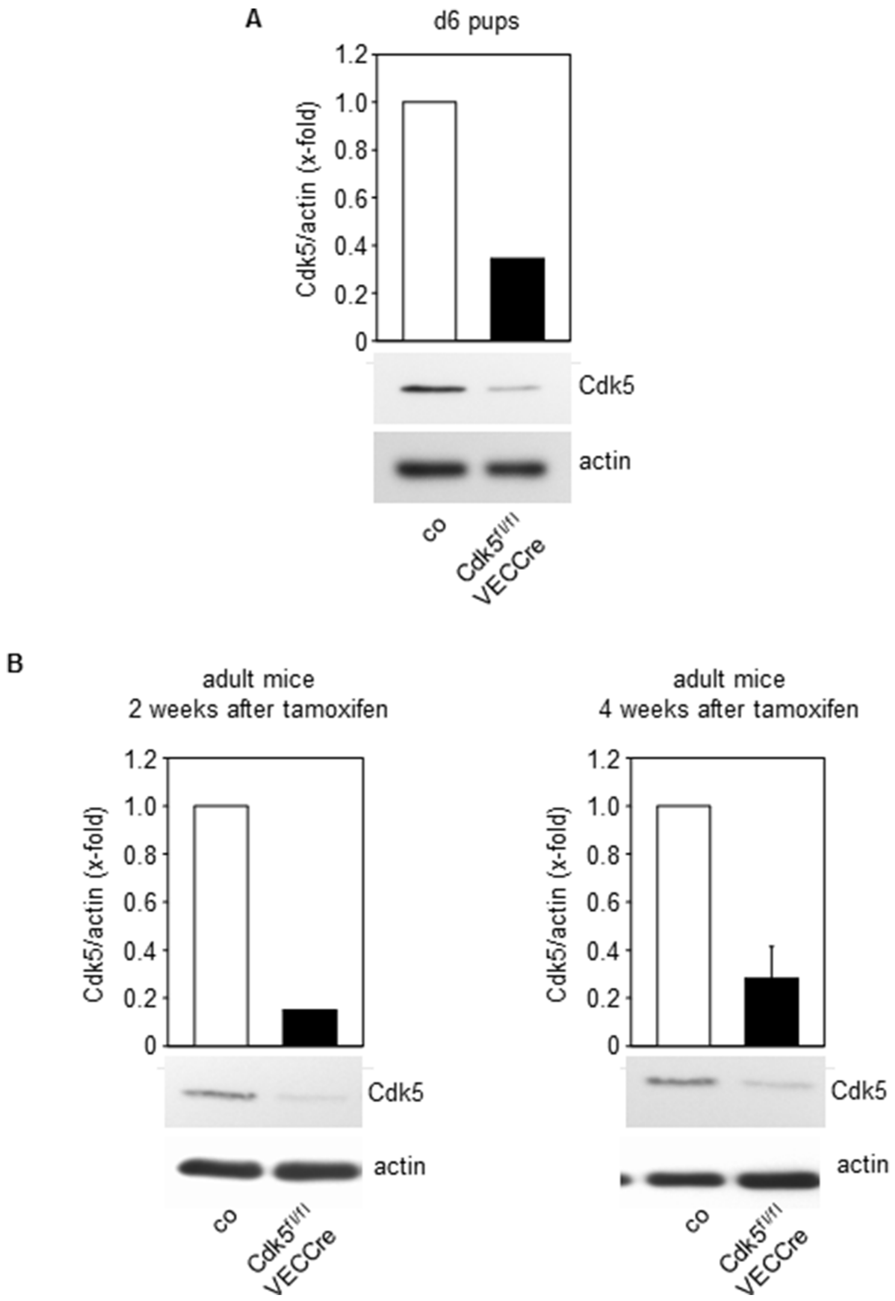

Supplementary Figure S1: Cdk5 Knockdown in inducible Cdk5$^{fl/fl}$VECCre mice. (**A**) Immunoblots and quantitative evaluation of liver sinusoidal endothelial cells (LSECs) of d6 pups with control and Cdk5$^{fl/fl}$VECCre genotype that were injected with tamoxifen (50 µg / day i.p.) from day 1 to day 3 for Cdk5 and actin (loading control) is shown (**B**) Western blots and quantitative evaluations of liver sinusoidal endothelial cells (LSECs) of adult mice with control and Cdk5$^{fl/fl}$VECCre genotype that were injected with tamoxifen (0.5 mg / day i.p.) for five consecutive days two weeks and four weeks after tamoxifen injection for Cdk5 and actin (loading control) are shown.
